# Effect of Phosphatidylserine and Cholesterol on Membrane-mediated Fibril Formation by the N-terminal Amyloidogenic Fragment of Apolipoprotein A-I

**DOI:** 10.1038/s41598-018-23920-3

**Published:** 2018-04-03

**Authors:** Chiharu Mizuguchi, Mitsuki Nakamura, Naoko Kurimitsu, Takashi Ohgita, Kazuchika Nishitsuji, Teruhiko Baba, Akira Shigenaga, Toshinori Shimanouchi, Keiichiro Okuhira, Akira Otaka, Hiroyuki Saito

**Affiliations:** 1 Department of Biophysical Chemistry, Kyoto Pharmaceutical University, 5 Nakauchi-cho, Misasagi, Yamashina-ku, Kyoto, 607-8414 Japan; 2 Graduate School of Pharmaceutical Sciences, Tokushima University, 1-78-1 Shomachi, Tokushima, 770-8505 Japan; 3 Department of Molecular Pathology, Institute of Biomedical Sciences, Tokushima University Graduate School, 3-18-15 Kuramoto-cho, Tokushima, 770-8503 Japan; 4 Biotechnology Research Institute for Drug Discovery, National Institute of Advanced Industrial Science and Technology (AIST), Tsukuba, 305-8565 Japan; 5 Graduate School of Environmental and Life Science, Okayama University, Okayama, 700-8530 Japan

## Abstract

Here, we examined the effects of phosphatidylserine (PS) and cholesterol on the fibril-forming properties of the N-terminal 1‒83 fragment of an amyloidogenic G26R variant of apoA-I bound to small unilamellar vesicles. A thioflavin T fluorescence assay together with microscopic observations showed that PS significantly retards the nucleation step in fibril formation by apoA-I 1‒83/G26R, whereas cholesterol slightly enhances fibril formation. Circular dichroism analyses demonstrated that PS facilitates a structural transition from random coil to α-helix in apoA-I 1‒83/G26R with great stabilization of the α-helical structure upon lipid binding. Isothermal titration calorimetry measurements revealed that PS induces a marked increase in capacity for binding of apoA-I 1‒83/G26R to the membrane surface, perhaps due to electrostatic interactions of positively charged amino acids in apoA-I with PS. Such effects of PS to enhance lipid interactions and inhibit fibril formation of apoA-I were also observed for the amyloidogenic region-containing apoA-I 8‒33/G26R peptide. Fluorescence measurements using environment-sensitive probes indicated that PS induces a more solvent-exposed, membrane-bound conformation in the amyloidogenic region of apoA-I without affecting membrane fluidity. Since cell membranes have highly heterogeneous lipid compositions, our findings may provide a molecular basis for the preferential deposition of apoA-I amyloid fibrils in tissues and organs.

## Introduction

Apolipoprotein A-I (apoA-I) is the principal protein in plasma high-density lipoprotein (HDL) and plays a central role in the formation and metabolism of HDL particles^1,2^. Human apoA-I is a 243-residue polypeptide that folds into two tertiary structural domains, comprising an N-terminal highly α-helical domain (residues 1–189) and a more flexible C-terminal domain (residues 190–243)^3–5^. Naturally occurring mutations in human apoA-I are known to be associated with low plasma HDL concentrations and hereditary amyloidosis^6^, in which the amyloidogenic mutations are clustered in two segments of the N-terminal domain (residues 1–90 and 154–178)^7–9^. In most hereditary amyloidogenic mutations associated with familial amyloid polyneuropathy, the N-terminal 80–100-residue fragments of the mutated apoA-I are found to deposit as amyloid fibrils in peripheral organs such as kidneys, heart, liver, and gastrointestinal tract^10–12^. The proteolytic cleavage of the full-length protein and the release of these N-terminal fragments could potentially happen either prior or following the protein misfolding and aggregation of the amyloidogenic apoA-I variants^9^. To date, the molecular mechanisms of the onset and development of these pathologies are largely unknown.

In a cellular environment, interactions with cell membranes are thought to play a critical role in the aggregation and fibrillation of a variety of amyloidogenic proteins^13–15^. Membrane binding increases the local concentration of proteins and reduces the diffusion dimension to the cell surface plane, resulting in increase in the probability of molecular collision with other proteins to aggregate^16,17^. In some natively unstructured amyloidogenic proteins such as islet amyloid polypeptide (IAPP) and α-synuclein, the formation of partially α-helical conformers upon membrane binding facilitates the aggregation of proteins through exposure of highly amyloidogenic sequences^18–20^. Alternatively, it is known that stabilization of the α-helical structure of proteins generates a kinetic trap in transition to β-strand structure, thereby inhibiting the aggregation and fibril formation^21–23^. In apoA-I, the N-terminal amino acid 1‒83 or 1‒93 fragments that are predominantly a random coil structure in solution have a strong propensity to form amyloid fibrils^11,24–26^. However, a lipid environment promotes the transition to the α-helical structure, thereby preventing the β-aggregation and fibril formation of the proteins^27,28^. Interestingly, the G26R mutation, the first and most common amyloidogenic mutation found in apoA-I^10,29^, was shown to facilitate fibril formation of the N-terminal 1‒83 fragment of apoA-I on membrane surfaces through a partial destabilization of α-helical conformation^28^.

Cell membranes have highly heterogeneous lipid compositions that vary between cell types and organelles^30,31^. Such variety of lipid composition in cell membranes is crucial in modulating the interactions of amphipathic proteins with membranes. In particular, negatively charged phospholipid (PL) such as phosphatidylserine (PS) and phosphatidylglycerol are well known to influence the kinetics of the aggregation and fibril formation of many amyloidogenic proteins on the membrane surface^27,32–36^. In addition, neutral lipid components such as cholesterol and phosphatidylethanolamine were shown to regulate the membrane interaction and fibril formation of α-synuclein^32,37,38^ and IAPP^36,39,40^. Although the effects of lipid composition on the interaction of apoA-I with lipid membranes have been extensively studied^41–44^, little is known about modulation of the kinetics of the fibrillogenic process of apoA-I by different membrane compositions.

In the present study, we investigated the effects of PS and cholesterol on the fibril-forming properties of the N-terminal 1‒83 fragment of the G26R variant of apoA-I bound to small unilamellar vesicles (SUVs). We also examined the modulation of lipid interaction and structural transition of apoA-I upon membrane binding by PS and cholesterol. The results indicate that PS strongly inhibits fibril formation of the N-terminal 1‒83 fragment of apoA-I G26R variant through stabilization of α-helical structure on the membrane environment.

## Results

### Effects of PS and cholesterol on fibril formation of apoA-I 1‒83/G26R on SUV

**Figure 1:**
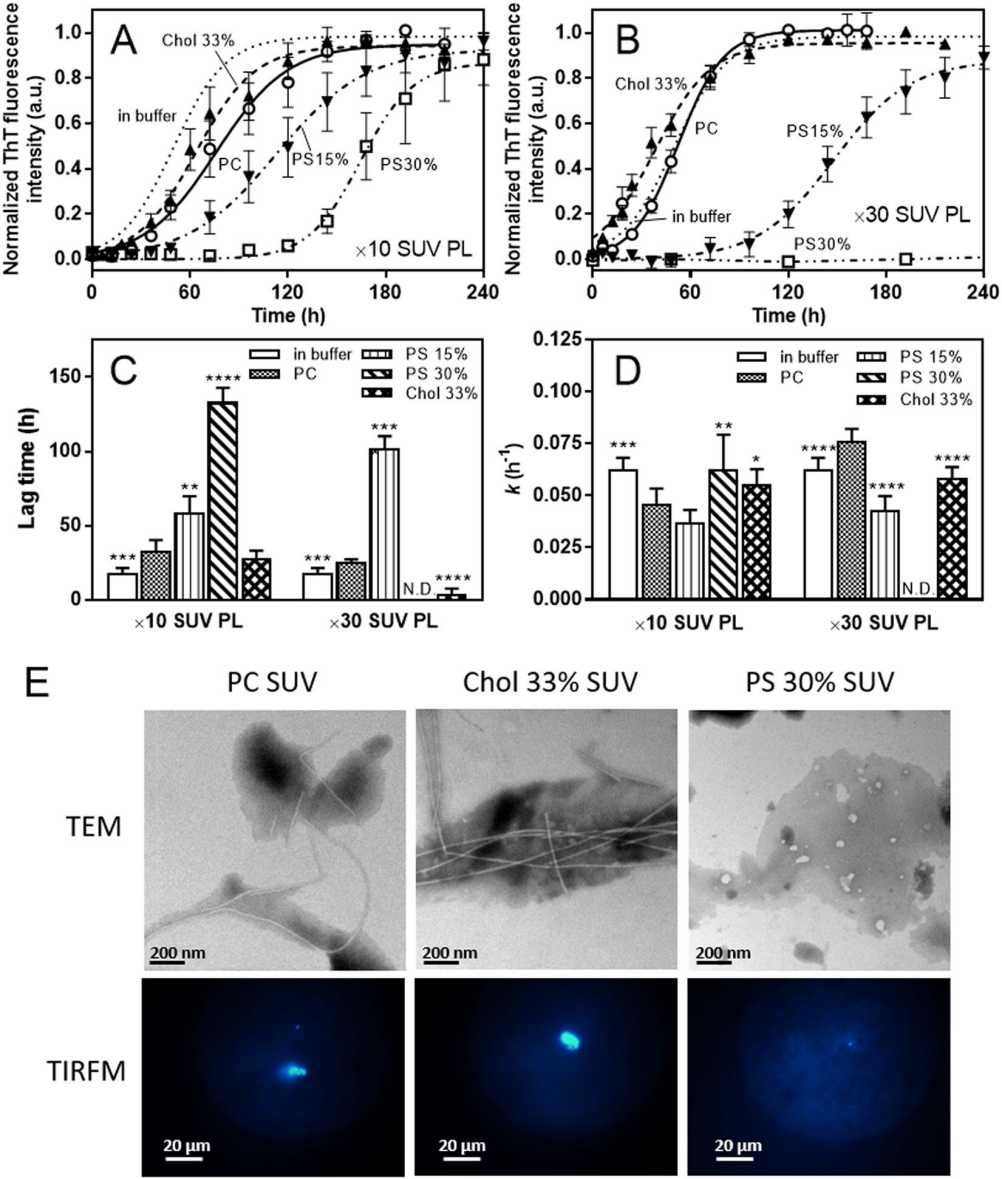
Effects of PS and cholesterol on formation of amyloid-like structure of apoA-I 1‒83/G26R bound to SUV monitored by ThT fluorescence. (**A,B**) PL/apoA-I weight ratios were 10 (**A**) and 30 (**B**). Dotted line, in buffer; ○, PC SUV; ▲, PC/Chol (2/1) SUV; ▼, PC/PS (17/3) SUV; □, PC/PS (7/3) SUV. ApoA-I 1‒83 variants were incubated at 37 °C with agitation on an orbital rotator in the presence of 10 μM ThT. Protein concentration was 0.05 mg/ml. *a*. *u*., arbitrary units. (**C**,**D**) Comparison of lag time (**C**) and apparent rate constant (**D**) for the fibril growth of apoA-I 1‒83/G26R bound to SUV. N.D., not determined. *p < 0.05; **p < 0.01; ***p < 0.001; ****p < 0.0001 *versus* “PC”. (**E**) TEM and TIRFM images of apoA-I 1‒83/G26R after incubation for 120 h in the presence of SUV at PL/apoA-I weight ratio of 10. Scale bars indicate 200 nm and 20 μm, respectively.

We previously demonstrated that although membrane binding greatly inhibits fibril formation by apoA-I 1‒83 fragment, the G26R variant retains the ability to form fibrils on the membrane surface^28^. Therefore, we first assessed the effects of PS and cholesterol on the fibril-forming properties of apoA-I 1‒83/G26R in the presence of phosphatidylcholine (PC) SUV using the amyloidophilic fluorescent dye, thioflavin T (ThT). As shown in Fig. 1A and B, apoA-I 1‒83/G26R exhibited sigmoidal increases in ThT fluorescence with increasing time in solution as well as on lipid membranes. Such time-dependent increase in ThT fluorescence gradually retarded with increasing contents of PS in PC SUV, and essentially no increase in ThT fluorescence was observed for PC/PS (7/3) SUV at PL to apoA-I weight ratio of 30 (Fig. 1B). In contrast, addition of cholesterol slightly accelerated the increase in ThT fluorescence for apoA-I 1‒83/G26R on PC SUV. Comparison of kinetic parameters for fibril formation by apoA-I 1‒83/G26R shows that addition of PS greatly increases the lag time without significantly affecting the apparent rate constant for the fibril growth (Fig. 1C and D). This indicates that PS greatly retards the nucleation step for fibril formation by apoA-I 1‒83/G26R on the membrane surface. We note that no significant increases in ThT fluorescence were observed for wild-type apoA-I 1–83 fragment in the presence of SUVs containing PS or Chol (Fig. S1).

Figure 1E shows transmission electron microscopy (TEM) and total internal reflection fluorescence microscopy (TIRFM) images of apoA-I 1‒83/G26R after incubation for 120 h in the presence of various SUVs. The formation of ThT-active, straight fibrils by the 1‒83/G26R was observed on both PC and PC/Chol (2/1) SUVs whereas no apparent fibrils were found for PC/PS (7/3) SUV, further indicating that PS greatly inhibits fibril formation by apoA-I 1‒83/G26R on lipid membranes. It should be noted that there was no alteration in the protein integrity upon incubation (Fig. S2).

### Effects of PS and cholesterol on membrane-binding properties of apoA-I 1‒83/G26R

**Figure 2:**
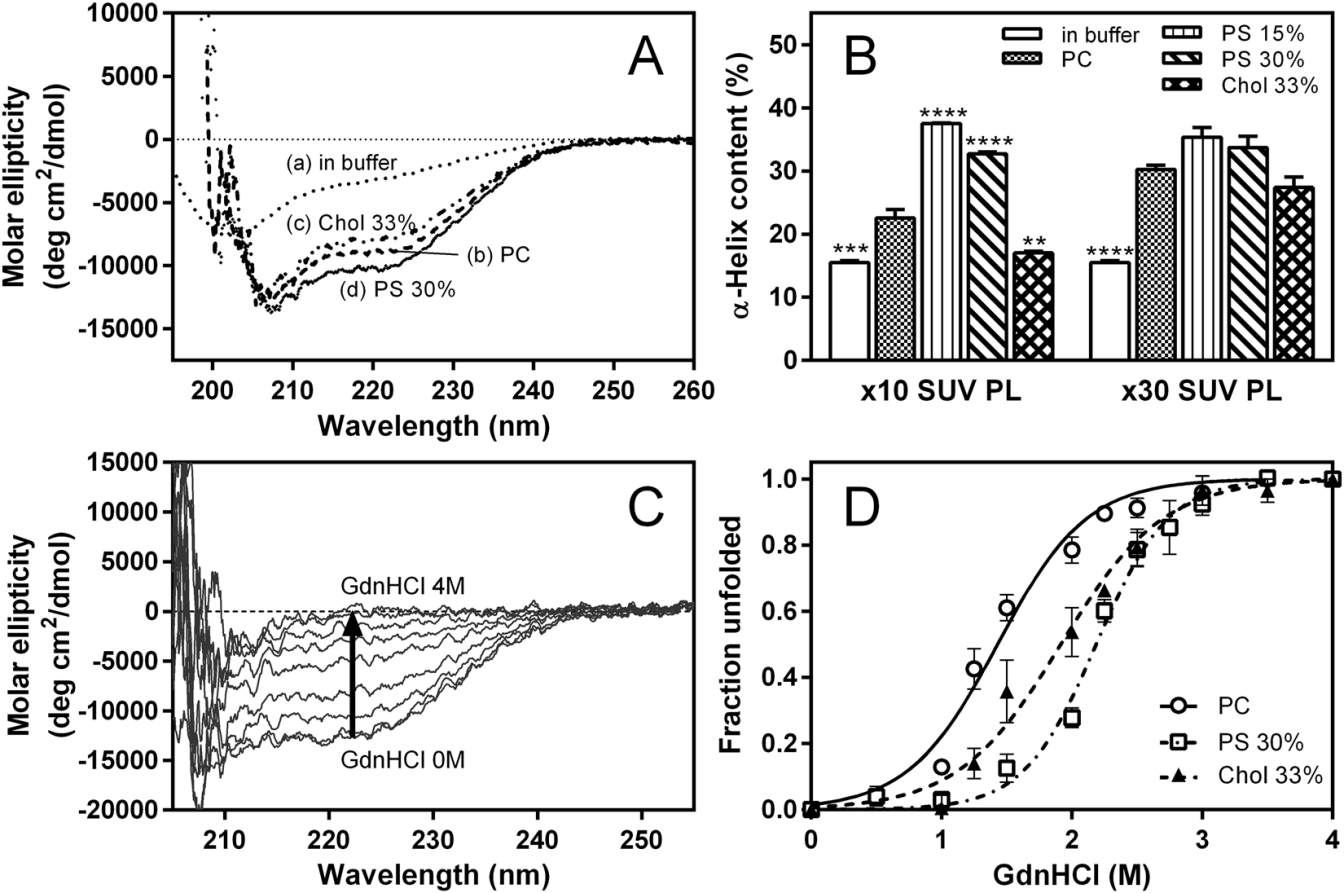
Formation and stability of α-helical structure of apoA-I 1‒83/G26R upon binding to SUV. (**A**) CD spectra of apoA-I 1‒83/G26R in buffer (a), in the presence of PC (b), PC/Chol (2/1) (c), or PC/PS (7/3) (d) SUVs. Protein and PL concentrations were 0.05 and 1.5 mg/ml, respectively. (**B**) Comparison of α-helix contents of apoA-I 1‒83/G26R in buffer or bound to SUV with the weight ratios of PL to apoA-I of 10 and 30. **p < 0.01; ***p < 0.001; ****p < 0.0001 *versus* “PC”. (**C**) Changes in CD spectra of apoA-I 1‒83/G26R bound to PC/PS (7/3) SUV upon increasing concentrations of GdnHCl. (**D**) GdnHCl denaturation curves of apoA-I 1‒83/G26R bound to PC (○), PC/Chol (2/1) (▲), or PC/PS (7/3) (□) SUVs.

We next examined the effects of PS and cholesterol on the secondary structure and stability of apoA-I 1‒83/G26R on the SUV surface. Far-UV circular dichroism (CD) spectra demonstrated that apoA-I 1‒83/G26R undergoes a structural transition from predominantly random coil to α-helix upon SUV binding^28^ (Fig. 2A). Comparison of α-helix contents derived from the CD spectra for apoA-I 1‒83/G26R bound to various SUVs indicates that addition of PS significantly enhances the α-helix formation of apoA-I 1‒83/G26R upon SUV binding, whereas cholesterol tends to decrease the α-helix contents (Fig. 2B). Figure 2C shows far-UV CD spectra of apoA-I 1‒83/G26R bound to PC/PS (7/3) SUV with increasing concentrations of guanidine hydrochloride (GdnHCl). The fraction of apoA-I unfolded at a given GdnHCl concentration derived from the changes in molar ellipticity at 222 nm^45^ was plotted as a function of GdnHCl concentration (Fig. 2D). Apparently, the denaturation curve of apoA-I 1‒83/G26R bound to SUV was shifted to higher concentration of GdnHCl by the presence of PS or cholesterol. Comparison of thermodynamic parameters of denaturation demonstrates great increases in the free energy of denaturation for PS or cholesterol-containing SUVs (Table 1), indicating that PS as well as cholesterol significantly increase the stability of α-helix structure of apoA-I 1‒83/G26R on the membrane surface.

**Table 1:** Parameters of GdnHCl-induced denaturation of apoA-I 1‒83/G26R bound to SUVs^a^.

SUV	Δ*G*_D_°	*D*_1/2_	*m*
	*kcal/mol*	*M*	*kcal/mol apoA-I/mol GdnHCl*
PC	2.4 ± 0.7	1.5 ± 0.1	1.6 ± 0.5
PC/Chol (2/1)	3.3 ± 0.4	2.0 ± 0.2	1.7 ± 0.3
PC/PS (17/3)	2.9 ± 0.6	1.9 ± 0.2	1.9 ± 0.1
PC/PS (7/3)	4.4 ± 0.2	2.1 ± 0.1	2.1 ± 0.1

^a^The data were from three independent experiments.

**Figure 3:**
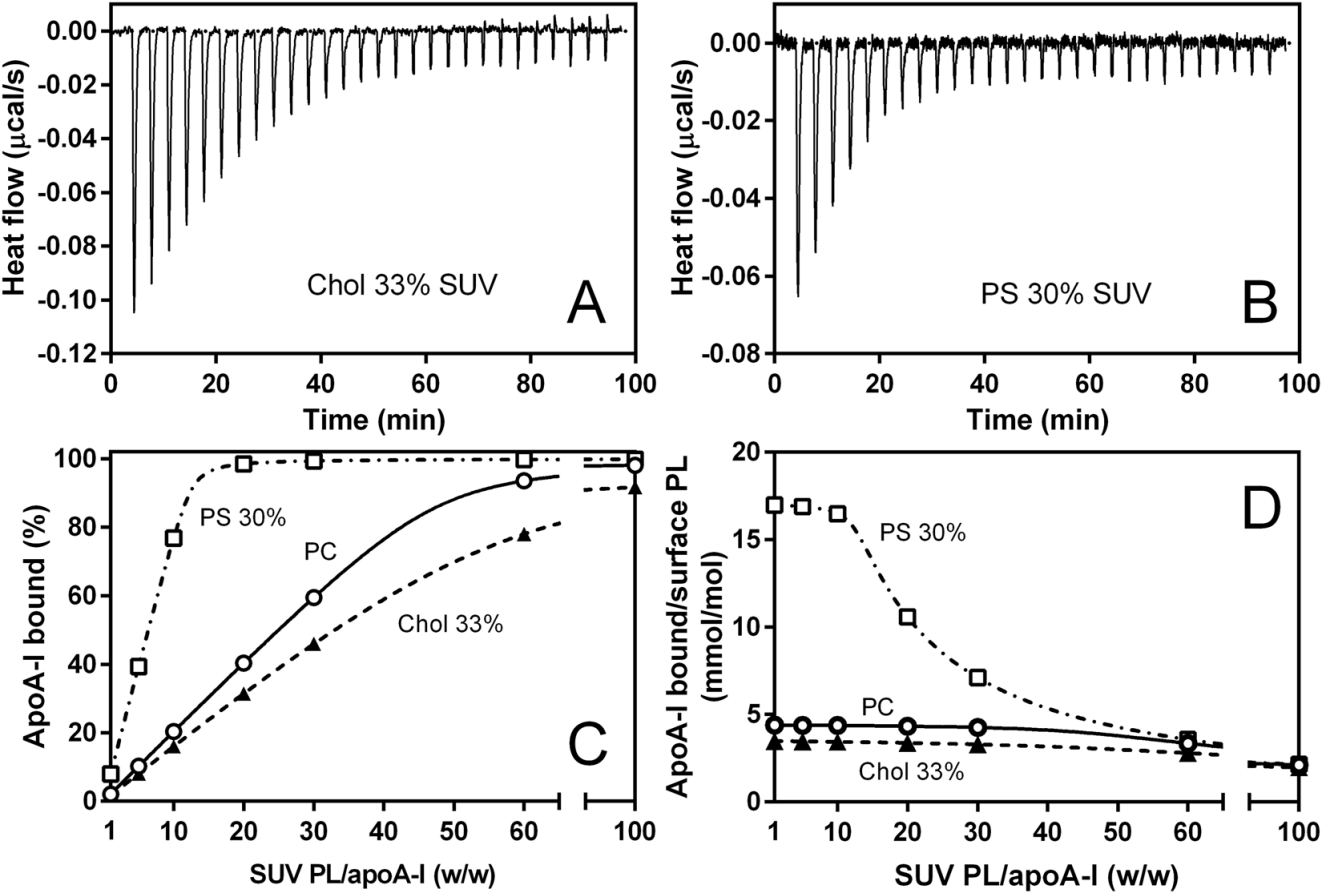
ITC for binding of apoA-I 1‒83/G26R to SUV. (**A,B**) Isothermal titration thermograms for binding of apoA-I 1‒83/G26R to PC/Chol (2/1) (**A**) and PC/PS (7/3) (**B**) SUVs. (**C,D**) Changes in the fraction percent of apoA-I 1‒83/G26R bound to SUV (**C**) and molar ratio of bound apoA-I to surface PL on SUV (**D**) as a function of weight ratio of PL to apoA-I. ○, PC SUV; ▲, PC/Chol (2/1) SUV; □, PC/PS (7/3) SUV. Fraction percent of apoA-I bound to SUV were calculated from *K*_d_ and *B*_max_ values listed in Table 2. Molar ratios of bound apoA-I to surface PL on SUV were calculated by assuming that surface PL on the outer layer of SUV available for binding of apoA-I is 67% of total PL.

To further investigate the effects of PS and cholesterol on the membrane-binding properties of apoA-I 1‒83/G26R, isothermal titration calorimetry (ITC) measurements were employed. Measurements of dynamic light scattering indicated that there was no change in size distribution of SUV upon binding of apoA-I (Fig. S3). Figure 3A and B show titration curves for binding of apoA-I 1‒83/G26R to PC/Chol (2/1) and PC/PS (7/3) SUVs. Binding of apoA-I 1‒83/G26R to SUV was accompanied with large exothermic heats, indicating an enthalpically favorable process^28^. The obtained thermodynamic parameters of binding are listed in Table 2. Addition of PS greatly increased the binding capacity and reduced the favorable binding enthalpy, whereas addition of cholesterol somewhat reduced the binding affinity and increased the favorable binding enthalpy. These results suggest that apoA-I 1‒83/G26R binds to PS-containing membranes with a mechanism distinct from that of PC or PC/Chol membranes.

**Table 2:** Thermodynamic parameters of binding of apoA-I 1‒83/G26R to SUVs at 25 °C^a^.

SUV	*K*_d_ (μg/ml)	*B*_max_ (amino acids/mol PL)	Δ*G*^b^ (kcal/mol)	Δ*H* (kcal/mol)	*T*Δ*S*^c^ (kcal/mol)
PC	1.1 ± 0.5	0.14 ± 0.03	−11.9 ± 0.3	−20.3 ± 0.8	−8.4 ± 0.8
PC/Chol (2/1)	3.8 ± 0.4	0.12 ± 0.02	−11.1 ± 0.1	−24.6 ± 0.1	−13.5 ± 0.1
PC/PS (17/3)	1.0 ± 0.9	0.28 ± 0.07	−12.1 ± 0.6	−12.4 ± 0.1	−0.3 ± 0.6
PC/PS (7/3)	0.5 ± 0.4	0.53 ± 0.19	−11.6 ± 0.1	−11.6 ± 0.1	0.9 ± 0.5

^a^The data were from three independent experiments.

^b^Free energy was calculated according to Δ*G* = *−RT* ln 55.5(1/*K*_d_).

^c^The entropy of binding was calculated from Δ*G* = Δ*H* − *T*Δ*S*.

Based on the dissociation constant, *K*_d_, and maximal binding capacity, *B*_max_, values for the SUV binding of apoA-I 1‒83/G26R listed in Table 2, we estimated the fraction and amount of apoA-I bound to various SUVs at different PL/apoA-I ratios. Figure 3C and D compare percentages of apoA-I bound to various SUVs and surface concentrations of apoA-I on the SUV surface, respectively, with increasing PL/apoA-I ratios. At low PL/apoA-I ratio of 10, PC/PS (7/3) SUV surfaces are saturated with apoA-I 1‒83/G26R (Fig. 3C) and the amount of apoA-I bound to the SUV surface is about 3 times higher than that to PC or PC/Chol (2/1) SUV (Fig. 3D). In contrast, at high PL/apoA-I ratios (>30), the PC SUV surface becomes saturated with apoA-I 1‒83/G26R (Fig. 3C) and the amounts of bound apoA-I are similar for PC, PC/PS (7/3), and PC/Chol (2/1) SUVs (Fig. 3D). Thus, the impaired fibril formation of apoA-I 1‒83/G26R on the PS-containing SUV surface is not likely due to the low surface concentration of apoA-I. Rather, it is conceivable that the increased propensity to form α-helix upon binding to PS-containing SUV inhibits aggregation and fibril formation of apoA-I 1‒83/G26R on the membrane surface.

### Effects of PS and cholesterol on membrane-binding and fibril-forming properties of amyloidogenic prone region-containing apoA-I peptides

**Figure 4:**
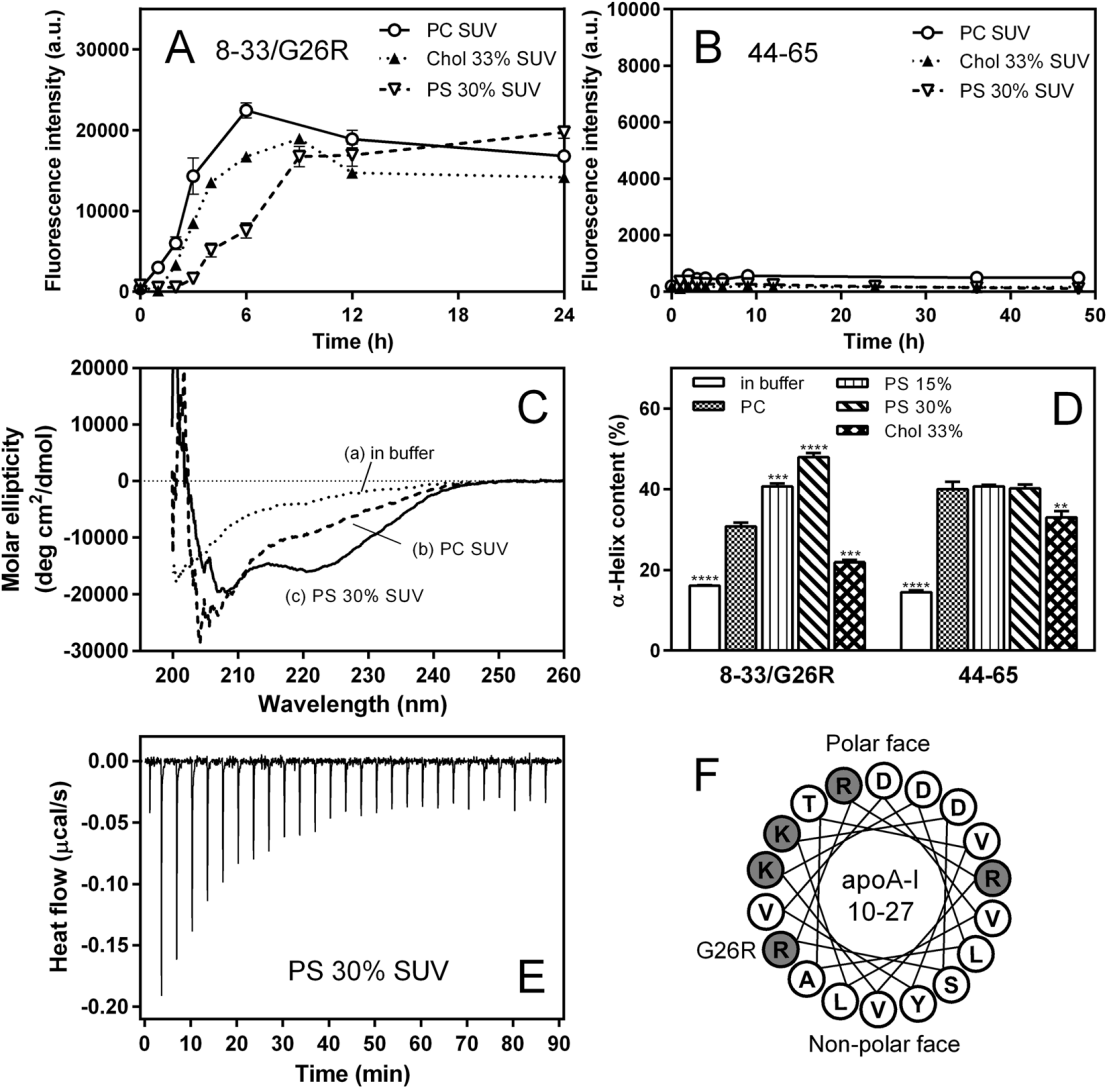
Fibril-forming and membrane-binding properties of apoA-I fragment peptides corresponding to the N-terminal amyloidogenic regions. (**A,B**) Formation of amyloid-like structure was monitored by ThT fluorescence for apoA-I 8‒33/G26R (**A**) and 44‒65 (**B**) peptides incubated at pH 7.4 in the presence of PC (○), PC/Chol (2/1) (▲), or PC/PS (7/3) (∇) SUVs. ApoA-I peptides were incubated at 37 °C with agitation on microplate shaker in the presence of 10 μM ThT. Peptide and PL concentrations were 0.1 and 6.0 mg/ml, respectively. *a*. *u*., arbitrary units. (**C**) CD spectra of apoA-I 8‒33/G26R in buffer (a), in the presence of PC (b), or PC/PS (7/3) (c) SUVs. Peptide and PL concentrations were 0.05 and 3.0 mg/ml, respectively. (**D**) α-Helix contents of apoA-I 8‒33/G26R and 44‒65 peptides in buffer or bound to SUVs. **p < 0.01; ***p < 0.001; ****p < 0.0001 *versus* “PC”. (**E**) Isothermal titration thermogram for binding of apoA-I 8‒33/G26R to PC/PS (7/3) SUV. (**F**) Helical wheel diagram of residues 10‒27 of apoA-I 1‒83/G26R.

We next examined the effects of PS and cholesterol on the membrane-binding and fibril-forming properties of apoA-I 8‒33/G26R and 44–65 peptides. ApoA-I 8‒33/G26R and 44–65 peptides contain the first (residues 14‒22) and the second (residues 49‒57) amyloidogenic segments in the N-terminal 1‒83 residues of apoA-I, respectively^46,47^. ThT fluorescence assay demonstrated that the 8‒33/G26R peptide has a strong ability to form amyloid-like structure on the SUV surface^28^, and the presence of PS significantly retards the kinetics of fibril formation (Fig. 4A). In contrast, no significant development of ThT fluorescence was observed for the 44‒65 peptide bound to any SUV surfaces (Fig. 4B).

CD measurements revealed that the 8–33/G26R peptide undergoes the transition from random coil to α-helix upon SUV binding, with the presence of PS greatly enhancing the propensity to form α-helix (Fig. 4C). Comparison of the α-helix contents of the 8‒33/G26R and 44‒65 peptides upon binding to various SUVs (Fig. 4D) clearly demonstrates that the α-helix formation of the 8‒33/G26R peptide upon SUV binding is significantly enhanced by the presence of PS, but is reduced by cholesterol. In contrast, enhanced α-helix formation upon binding to PS-containing SUV was not seen for the 44‒65 peptide, indicating that residues 8‒33/G26R contain the region responsible for the PS-induced α-helix formation of apoA-I 1‒83/G26R fragment. As shown in Fig. 4E, ITC measurements demonstrated that binding of the 8‒33/G26R peptide to PC/PS (7/3) SUV is an exothermic process similar to the case of the 1‒83/G26R (Fig. 3B), and the 8‒33/G26R binds to PC/PS (7/3) SUV with greatly increased binding capacity and affinity compared to PC SUV (Table 3). The helical wheel diagram of residues 10‒27 in apoA-I 1‒83/G26R (Fig. 4F) indicates that positively charged arginine and lysine residues including R26 are largely located at the polar-nonpolar interface. Thus, it is possible that positively charged residues in residues 10‒27 of apoA-I 1‒83/G26R interact electrostatically with negatively charged PL at the membrane interfacial region^48^.

**Table 3:** Thermodynamic parameters of binding of apoA-I 8‒33/G26R peptide to SUVs at 25 °C^a^.

SUV	*K*_d_ (μg/ml)	*B*_max_ (amino acids/mol PL)	Δ*G*^b^ (kcal/mol)	Δ*H* (kcal/mol)	*T*Δ*S*^c^ (kcal/mol)
PC	2.9 ± 1.3	0.03 ± 0.01	−10.7 ± 0.3	−13.8 ± 0.2	−3.2 ± 0.5
PC/PS (7/3)	0.8 ± 0.1	0.11 ± 0.01	−11.2 ± 0.1	−16.2 ± 0.4	−4.9 ± 0.1

^a^The data were from two or three independent experiments.

^b^Free energy was calculated according to Δ*G* = −*RT* ln 55.5(1/*K*_d_).

^c^The entropy of binding was calculated from Δ*G* = Δ*H* − *T*Δ*S*.

### Evaluation of membrane-bound states of apoA-I 1‒83/G26R variants using fluorescent probes

**Figure 5:**
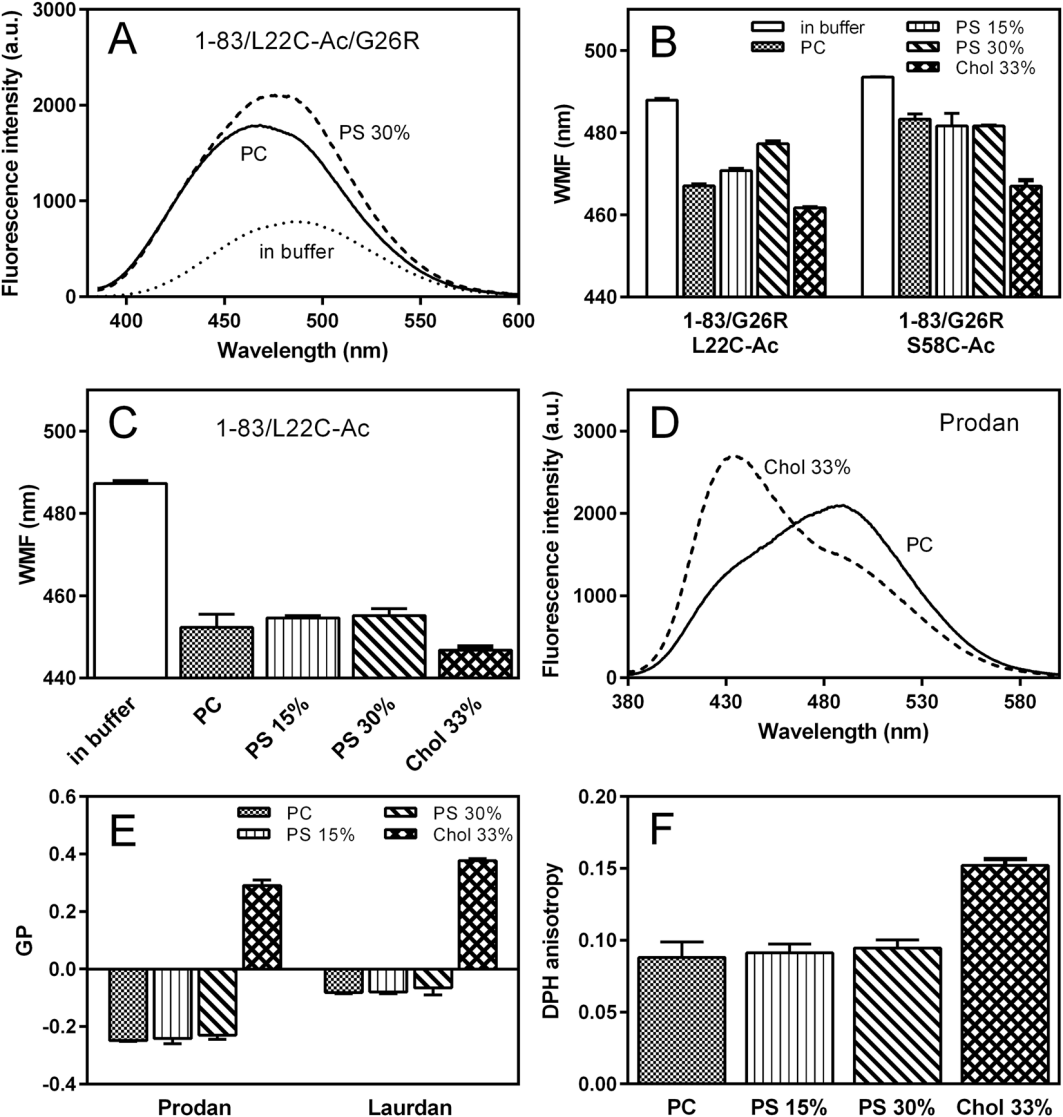
Effects of PS and cholesterol on membrane interactions of apoA-I 1‒83/G26R. **(A)** Fluorescence emission spectra of acrylodan in apoA-I 1‒83/L22C-Ac/G26R in the absence or presence of PC or PC/PS (7/3) SUVs. *a*. *u*., arbitrary units. **(B)** Changes in WMF of apoA-I 1‒83/G26R variants labeled with acrylodan by binding to various SUVs. **(C)** Changes in WMF of apoA-I 1‒83/L22C-Ac by binding to various SUVs. **(D)** Fluorescence emission spectra of prodan in PC or PC/Chol (2/1) SUVs. *a*. *u*., arbitrary units. **(E)** GP of prodan and laurdan in various SUVs. **(F)** Fluorescence anisotropy of DPH in various SUVs.

We further investigated the effects of PS and cholesterol on the lipid-bound conformation of apoA-I 1‒83/G26R using site-specific acrylodan (Ac) labeling of apoA-I. For attachment of acrylodan to apoA-I 1‒83/G26R, we introduced Cys mutations, L22C or S58C, around the first (residues 14‒22) and the second (residues 49‒57) amyloidogenic regions in the N-terminal 1‒83 residues of apoA-I, respectively. We note that these Cys mutations do not largely perturb the lipid-binding properties of apoA-I 1‒83/G26R (Fig. S4), similar to the case of full-length apoA-I^49,50^. Figure 5A shows fluorescence emission spectra of acrylodan in apoA-I 1‒83/L22C-Ac/G26R in solution and bound to PC or PC/PS (7/3) SUVs. Significant blue shifts in wavelength of maximum fluorescence (WMF) upon SUV binding were observed, indicating that the acrylodan molecule is embedded in a hydrophobic lipid environment^28,51,52^. Comparison of the WMF of the acrylodan-labeled 1‒83/G26R variants bound to SUV (Fig. 5B) indicates that on the SUV surface, addition of PS increases the solvent exposure of acrylodan attached at position 22, but has no effect on the environment of acrylodan at position 58. In contrast, cholesterol tends to induce a more hydrophobic environment at both positions 22 and 58. Interestingly, such an effect of PS on the environment of acrylodan at position 22 was not observed in the apoA-I 1‒83/L22C-Ac variant (Fig. 5C). These results indicate that the presence of PS induces a more solvent-exposed conformation around the first amyloidogenic region in apoA-I 1‒83/G26R, in which residue R26 is responsible for the interaction with PS.

To check whether PS increases the binding of apoA-I to the membrane surface^53^ by altering the membrane fluidity, we measured the generalized polarization (GP) of prodan and laurdan, fluorescent membrane probes that have high sensitivity to the polarity of the environment^54,55^. Prodan and laurdan are located at the interfacial region of lipid membranes, with the fluorescent moiety of laurdan residing in a more hydrophobic region than prodan^56^. As shown in Fig. 5D, fluorescence emission spectra of prodan for PC/Chol (2/1) SUV exhibited a significant increase in the peak at around 430 nm and a concomitant decrease in the peak at around 490 nm compared to that for PC SUV, indicating that cholesterol induces more ordered lipid packing at the membrane surface^57^. Comparison of GP for prodan and laurdan in various SUVs demonstrates that compared to high positive values of GP for PC/Chol (2/1) SUV which indicate more ordered lipid packing, PC and PC/PS (7/3) SUVs exhibit similar negative values of GP (Fig. 5E). In addition, measurements of the fluorescence anisotropy of DPH that reflects molecular packing and fluidity of the acyl chain region in bilayer membranes demonstrated that addition of PS does not affect DPH anisotropy in SUV whereas cholesterol greatly increases DPH anisotropy (Fig. 5F), consistent with more ordered lipid packing in cholesterol-containing membranes^57^. Taken together, these results indicate that the presence of PS does not alter lipid packing and fluidity at the membrane surface, whereas cholesterol strongly induces ordered membrane packing.

## Discussion

So far, there are about 20 naturally occurring mutations in human apoA-I associated with hereditary systemic amyloidosis^9^. In most cases, the N-terminal 80–100-residue fragment is the predominant form of the mutated apoA-I found in amyloid fibril deposits. Although it is known that a preferential fibril deposition in certain tissues and organs occurs for some amyloidogenic variants of apoA-I^58^, the molecular basis for the preferential localization of amyloid fibrils is unclear.

In the present study, we found that PS strongly inhibits fibril formation by the 1‒83 fragment of the amyloidogenic G26R variant of apoA-I on membrane surfaces (Fig. 1). In a cellular environment, membrane lipid compositions as well as extracellular matrix components are thought to mediate formation and deposition of amyloid fibrils in many amyloidogenic proteins^59–61^. Indeed, glycosaminoglycans such as heparin and heparan sulfate were shown to play critical roles in formation and cellular interaction of apoA-I amyloid fibrils^26,62–64^. In addition, it has been demonstrated that membrane binding strongly inhibits fibril formation by the N-terminal fragments of apoA-I through entrapping the protein in a stable α-helical structure^27,28^. Since apoA-I predominantly exists in an α-helical conformation bound to HDL particles in plasma, HDL binding would prevent amyloid fibril formation of apoA-I *in vivo*. A recently study demonstrated that the HDL binding blocks fibril formation by serum amyloid A, an acute-phase apolipoprotein that causes systemic amyloidosis^65^. Interestingly, the G26R variant of apoA-I 1‒83 fragment can form amyloid fibrils on lipid membranes because of partially destabilized α-helical conformation^28^, indicating the importance of the balance between stabilized and destabilized α-helical conformations for protein aggregation. Consistent with this notion, the present results demonstrate that PS greatly enhances the formation and stabilization of the α-helical structure of apoA-I 1‒83/G26R variant upon membrane binding (Fig. 2 and Table 1), thereby preventing the transition to β-structure and aggregation. ITC (Fig. 3 and Table 2) and acrylodan fluorescence (Fig. 5A and B) measurements of the apoA-I 1‒83/G26R variants indicate that the enhanced α-helix formation of apoA-I by PS is accompanied by a greatly increased binding capacity and a more solvent-exposed conformation around the first amyloidogenic prone region (residues 14‒22) in apoA-I. Given a recent study reported that the N-terminal 1–93 fragment of apoA-I interacts with PC membranes in proximity to the lipid-water interface^66^, positively charged residues around the amyloidogenic region in apoA-I are likely to electrostatically interact with negatively charged PS at the membrane interfacial region. To support this idea, the inhibitory effects of PS on the fibril-forming ability of apoA-I 1–83/G26R on the SUV surface were enhanced by lowering solvent ionic strength (Fig. S5). Since PS did not affect the acrylodan environment in the poA-I 1‒83/L22C-Ac variant (Fig. 5C) and the membrane-binding properties of apoA-I 8‒33 peptide (Fig. S6 and Table S1) on the SUV surface, the mutated R26 residue is likely to be responsible for the interaction of apoA-I with PS.

PS as well as negatively charged PLs have been reported to induce aggregation and formation of fibril structure for a variety of amyloidogenic proteins^33,35,36,67^, suggesting that PS could provide the physiological low-pH environment on cellular membranes so as to enhance protein fibril formation^33^. It is known that PLs are distributed across plasma membranes of eukaryotic cells in an asymmetric manner, in which PS is predominantly present in the cytoplasmic leaflet^68^. However, PS exists on the surface of extracellular vesicles released from a variety of cells such as activated T-cells, platelets, and hypoxia-induced stem cells^69–71^. PS on these extracellular vesicles interacts with receptors for PS on the surface of various cells including phagocytes, endothelial cells, dendritic cells, and epithelial cells, thereby inducing cellular uptake of the extracellular vesicles^72,73^. Importantly, extracellular vesicles such as exosomes reportedly affect the formation of amyloid fibrils by some amyloidogenic proteins such as α-synuclein^74^ and IAPP^75^. These lines of evidence together with our findings suggest that extracellular vesicles secreted from a certain type of cells can contribute to or regulate the pathology of amyloidoses.

In contrast to PS, the presence of cholesterol accelerates to some extent fibril formation of apoA-I 1‒83/G26R on membrane surfaces (Fig. 1). It was shown that upon membrane binding, the amphipathic α-helices of apoA-I penetrate into the interfacial and outer acyl chain region of fluid lipid bilayer membranes^41,76^. Since cholesterol increases molecular packing and decreases fluidity in lipid membranes (Fig. 5D–F), the penetration of amphipathic α-helices of apoA-I into cholesterol-containing membranes appears to be partially inhibited^77^, resulting in decreased α-helix formation of apoA-I (Figs 2B and 4D). Since residues 8–33 contain a highly hydrophobic segment that has the high lipid-binding ability in the amino acid sequence of apoA-I 1–83 fragment^28^, the effect of cholesterol on the α-helix formation of apoA-I 8–33/G26R appears more pronounced compared to apoA-I 1–83/G26R. However, increased membrane hydrophobicity in the presence of cholesterol would strengthen the van der Waals interactions between the nonpolar face of amphipathic α-helices of apoA-I and acyl chain region of lipid membranes, leading to increased stability of α-helical conformation (Fig. 2D). Thus, the slightly enhanced fibril-forming propensity of apoA-I 1‒83/G26R on cholesterol-containing membranes likely comes from these opposite effects of cholesterol on the formation and stabilization of apoA-I α-helical structure. It should be noted that a previous study of apoA-I 1–93 fragment reported that cholesterol induces formation of α-helical structure, thus affecting its aggregation and fibrillation^27^. This discrepancy of effects of cholesterol may come from the fact that the 1–93 fragment contains additional residues 84–93 (QEMSKDLEEV) which are rich in negatively charged amino acids at neutral pH, leading to the different protein-protein and lipid-protein interactions.

Cholesterol in plasma membranes has been implicated in cytotoxicity^78–80^ and fibril formation^81,82^ of several amyloidogenic proteins, possibly by regulating the membrane binding or insertion of the proteins. Indeed, Ji *et al*. showed that a low concentration of membrane cholesterol retains amyloid β (Aβ) at the cell surface in a β-sheet structure, whereas an increased concentration of membrane cholesterol results in membrane insertion of Aβ^81^, which is partially consistent with our findings. In the present study, we further reported a novel action of PS: that is, although PS enhances the interaction between lipid membranes and amyloidogenic apoA-I fragments, PS inhibits the fibril formation by enhancing the α-helix formation. Thus, our finding supports the idea that cell milieu such as lipid composition of the plasma membranes is one of the determinant of tissue-selective amyloidogenesis by apoA-I variants^83^. Since PS is also shown to be involved in cytotoxicity of Aβ^84^, elucidation of effects of PS on the cytotoxicity of apoA-I fibrils would be a future challenge.

In conclusion, the current study showed that PS strongly inhibits fibril formation of the N-terminal 1‒83 fragment of apoA-I G26R variant on membrane surfaces through stabilization of α-helical conformation. We also demonstrated that PS facilitates α-helix formation of the amyloidogenic core region of apoA-I 1‒83/G26R probably through electrostatic interactions between positively charged amino acids in apoA-I and the negatively charged head group in PS at the membrane surface. Since cell membranes have highly heterogeneous lipid compositions, it seems plausible that specific lipid compositions of cell membranes such as negatively charged PL and cholesterol content could regulate the formation and deposition of apoA-I amyloid fibrils in certain tissues and organs. Recently, it was reported that specific cell milieus affect conformation, aggregation propensity, and fibrillogenesis of amyloidogenic apoA-I variants^83^. Thus, analysis of the lipid composition of apoA-I amyloidosis patients carrying various amyloidogenic mutations would deepen the understanding of the role of lipid in the development or prevention of amyloidoses.

## Materials and Methods

### Preparation of recombinant apoA-I proteins and peptides

The N-terminal 1–83 fragment of apoA-I and its engineered variants with substitutions of L22C, G26R, L22C/G26R, and G26R/S58C were expressed in *E*. *coli* as thioredoxin fusion proteins and isolated and purified as previously described^25,28^. The apoA-I 8–33, 8‒33/G26R, and 44‒65 peptides were synthesized by the solid-phase method with Fmoc chemistry^28,47^. The N and C termini were capped with an acetyl group and an amide group, respectively. The apoA-I variants and peptides were dialyzed from 6 M GdnHCl solution with or without 1% β-mercaptoethanol into the appropriate buffer before use. Labeling of cysteine-containing apoA-I variants with acrylodan (6-acryloyl-2-dimethylaminonaphthalene, Thermo Fisher Scientific) was performed as described^28,52^.

### Preparation of SUV

SUV was prepared as previously described^28,85^ with slight modifications of the protocol. Briefly, a dried film of egg PC (Kewpie, Tokyo, Japan) with or without 1-palmitoyl-2-oleoyl PS (NOF, Tokyo, Japan) or cholesterol (Sigma-Aldrich) was hydrated in 10 mM Tris-HCl buffer (150 mM NaCl, 0.02% NaN_3_, pH 7.4), followed by sonication on ice under nitrogen. To separate any remaining large vesicles, the resulting samples were centrifuged in a Beckman MLA-55 rotor for 1.5 h at 15 °C at 40,000 rpm.

### CD spectroscopy

Far-UV CD spectra were recorded from 190 to 260 nm at 25 °C using a Jasco J-1500 spectropolarimeter (JASCO, Tokyo, Japan) as previously described^28^. The α-helical content was derived from the molar residue ellipticity at 222 nm ([*θ*]_222_) using the equation: % α-helix = [(−[*θ*]_222_ + 3000)/(36000 + 3000)] × 100^86^.

For monitoring chemical denaturation, SUV-bound apoA-I 1‒83/G26R at a concentration of 50 μg/ml was incubated overnight at 4 °C with GdnHCl at various concentrations. The equilibrium constant of denaturation, *K*_D_, at a given GdnHCl concentration was calculated from the change in [*θ*]_222_ determined by CD in 10 mM Tris-HCl buffer (pH 7.4). The Gibbs free energy of denaturation in the absence of denaturant, Δ*G*_D_°, the midpoint of denaturation, *D*_1⁄2_, and *m* value that reflects the cooperativity of denaturation in the transition region, were determined by the linear equation, Δ*G*_D_ = Δ*G*_D_° − *m*[GdnHCl], where Δ*G*_D_ = −*RT* ln *K*_D_^45^.

### ITC measurements

Heats of binding of apoA-I variants or peptides to SUV were measured with a VP-ITC or iTC200 instruments (Malvern) at as previously described^28^. The SUV suspension was placed in the sample cell (1.33 ml or 0.2 ml for VP-ITC or iTC200, respectively) and titrated with 8–10-μl aliquots of the apoA-I sample with continual stirring at 400 rpm. The resulting ITC data were fitted to the one-site binding model in Origin 7.1 (MicroCal) to obtain thermodynamic parameters of binding. Binding of apoA-I to lipid particles can be analyzed with assuming a single isotherm equilibrium and a finite number of discrete, equivalent, and non-interacting binding sites on the lipid surface, which is mathematically equivalent to the one-site or Langmuir binding model^49,87,88^. The maximal binding capacity, *B*_max_, is represented as the number of amino acids of bound apoA-I per mol of phospholipids.

### Fluorescence measurements

Fluorescence measurements were carried out with F-2700 or F-7000 fluorescence spectrophotometers (Hitachi High-Technologies, Tokyo, Japan) and an *F*_max_ fluorescence plate reader (Molecular Devices) at 25 °C. To assess the local environment of acrylodan attached at the Cys-containing apoA-I 1‒83 variants, acrylodan emission fluorescence was collected from 380 to 600 nm with an excitation of 360 nm. To assess the lipid packing of the membrane interfacial region of SUV, SUV samples were labeled with prodan or laurdan^53^ to yield a PL:probe molar ratio of 200:1. Fluorescence emission spectra of prodan and laurdan were collected with excitation wavelengths of 350 and 361 nm for laurdan and prodan, respectively. The GP value was calculated from the emission intensities using the equation GP = (*I*_B_ − *I*_R_)/(*I*_B_ + *I*_R_), where *I*_B_ and *I*_R_ are the emission intensities at the blue (430 nm) and red (490 nm) edges of the emission spectrum, respectively^55^. To assess the lipid packing and fluidity of the membrane acyl chain region, SUV samples were labeled with DPH (1,6-diphenylhexatriene, Thermo Fisher Scientific) to yield a PL:DPH molar ratio of 200:1. Fluorescence anisotropy of DPH was measured at 430 nm with an excitation wavelength of 360 nm. Fluorescence anisotropy, *r*, is given by *r* = (*I*_0−0_ − *G I*_0−90_)/(*I*_0−0_ + 2 *G I*_0−90_), where *I* signifies fluorescence intensity, subscript 0 and 90 indicate the direction of the plane of polarization of the polarizer and the analyzer, respectively, and *G* (=*I*_90−0_/*I*_90−90_) represents the compensating factor for the anisotropy sensitivity of the instrument^89^.

Kinetics of formation of amyloid-like structure were monitored using ThT as previously described^28^. ApoA-I 1‒83 variants (50 μg/mL) or apoA-I peptides (100 μg/mL) in 10 mM Tris-HCl buffer (150 mM NaCl, 0.02% NaN_3_, pH 7.4) were incubated at 37 °C with agitation on an orbital rotator (for apoA-I 1–83 variants) or microplate shaker (for apoA-I peptides) in the presence of 10 μM ThT. The increase in ThT fluorescence intensity with time was fitted to a sigmoidal equation^25,28^ to obtain the apparent rate constant, *k*, for the fibril growth and the time to 50% of maximal fluorescence, *t*_*m*_. The lag time is calculated as *t*_*m*_ − 2/*k*.

### TEM and TIRFM

The TEM and TIRFM was performed as previously described^28^.

### Statistical analysis

Data were analyzed via one-way analysis of variance, including the appropriate variables, followed by Dunnett’s test. Results were regarded as significant for *P* < 0.05.

## Electronic supplementary material

Supplementary Information
