# Supplementary material for: Effect of Phosphatidylserine and Cholesterol on Membrane-mediated Fibril Formation by the N-terminal Amyloidogenic Fragment of Apolipoprotein A-I
Source: Sci Rep. 2018 Apr 3;8:5497. doi: 10.1038/s41598-018-23920-3 (PMC5882889; doi:10.1038/s41598-018-23920-3)
Supplement: Supplementary file 1 — Supplementary Information [file 41598_2018_23920_MOESM1_ESM.docx]

**Supplementary Information**

**Effect of Phosphatidylserine and Cholesterol on Membrane-mediated Fibril Formation by the N-terminal Amyloidogenic Fragment of Apolipoprotein A-I**

Chiharu Mizuguchi^1,2^, Mitsuki Nakamura^1^, Naoko Kurimitsu^1^, Takashi Ohgita^1^, Kazuchika Nishitsuji^3^, Teruhiko Baba^4^, Akira Shigenaga^2^, Toshinori Shimanouchi^5^, Keiichiro Okuhira^2^, Akira Otaka^2^, and Hiroyuki Saito^1^

^1^ Department of Biophysical Chemistry, Kyoto Pharmaceutical University, 5 Nakauchi-cho, Misasagi, Yamashina-ku, Kyoto 607-8414, Japan

^2^ Graduate School of Pharmaceutical Sciences, Tokushima University, 1-78-1 Shomachi, Tokushima 770-8505, Japan

^3^ Department of Molecular Pathology, Institute of Biomedical Sciences, Tokushima University Graduate School, 3-18-15 Kuramoto-cho, Tokushima 770-8503, Japan

^4^ Biotechnology Research Institute for Drug Discovery, National Institute of Advanced Industrial Science and Technology (AIST), Tsukuba 305-8565, Japan

^5^ Graduate School of Environmental and Life Science, Okayama University, Okayama 700-8530, Japan

**This supplementary data consists of:**

Figures S1‒S6 and Table S1

**Figure S1. Formation of amyloid-like structure of apoA-I 1‒83 bound to SUV.** Formation of amyloid-like structure was monitored by ThT fluorescence for apoA-I 1‒83 in the presence of SUV. PL/apoA-I weight ratio was 30. ○, in buffer; □, PC SUV; ▲, PC/Chol (2/1) SUV; ∇, PC/PS (7/3) SUV. Protein concentration was 0.05 mg/ml. *a. u.*, arbitrary units.

**Figure S2. MALDI-TOF MS spectra of apoA-I 1-83/G26R before (A) and after (B) 120h incubation.** MALDI-TOF MS analysis was performed using a microflex instrument (Bruker Daltonics, Bremen, Germany) in the positive linear ion mode. Spectra were calibrated externally using a standard protein mixture (Protein calibration standard I and II, Bruker). The ion observed at m/z 9774.8‒9774.9 was estimated to be the protonated molecule (M + H)^+^: molecular mass of the recombinant apoA-I 1-83/G26R is calculated to be 9773.81.

**Figure S3. Particle size distributions of PC (A) and PC/PS (7/3) (B) SUVs determined by dynamic light scattering measurements on a Zetasizer Nano ZS (Malvern).** The data were represented as volume-based distributions.

**Figure S4. Far-UV CD spectra of apoA-I 1-83/G26R L22C (A and B) and S58C (C and D) variants bound to PC or PC/PS (7/3) SUVs.** Errors in the CD spectra of apoA-I 1-83/G26R bound to SUVs were shown as dotted lines.

**Figure S5. Effect of salt concentration on fibril-forming property of apoA-I 1-83/G26R bound to SUV.** Formation of amyloid-like structure was monitored by ThT fluorescence for apoA-I 1‒83/G26R in the presence of SUV at NaCl concentration of 50 mM. PL/apoA-I weight ratio was 10. ○, PC SUV; ∇, PC/PS (7/3) SUV. Protein concentration was 0.05 mg/ml. *a. u.*, arbitrary units. The data at NaCl concentration of 150 mM (dotted line) are also shown for comparison.

**Figure S6. Effects of PS on membrane-binding properties of apoA-I 8‒33 peptide.** (**A**) Comparison of α-helix contents of apoA-I 8‒33 and 8‒33/G26R peptides in buffer or bound to various SUVs. (**B**) Isothermal titration thermogram for binding of apoA-I 8‒33 peptide to PC/PS (7/3) SUV.

| **Table S1. Thermodynamic parameters of binding of apoA-I 8‒33 peptide to SUVs at 25 °C**^a^ | | | | | | | | |
| --- | --- | --- | --- | --- | --- | --- | --- | --- |
|  |  |  |  |  | |  | |  |
| SUV | *K*_d_  (μg/ml) | *B*_max_  (amino acids/  mol PL) |  | ∆*G*^b^ (kcal/mol) | | ∆*H* (kcal/mol) | | *T*∆*S*^c^ (kcal/mol) |
| PC | 2.7 ± 0.9 | 0.05 ± 0.02 |  | −10.7 ± 0.2 | | −21.1 ± 1.6 | | −10.4 ± 1.8 |
| PC/PS (7/3) | 1.0 ± 0.2 | 0.16 ± 0.01 |  | −11.2 ± 0.1 | | −22.4 ± 0.1 | | −11.1 ± 0.1 |
| ^a^ The data were from two or three independent experiments.  ^b^ Free energy was calculated according to ∆*G* = −*RT* ln 55.5(1/*K*_d_).  ^c^ The entropy of binding was calculated from ∆*G* = ∆*H* ‒ *T*∆*S* | | | | |  | |  | |
|  | | | | |  | |  | |
